# Boramidine: a boron-based photoacidic fluorophore†

**DOI:** 10.1039/d5cc02043c

**Published:** 2025-05-15

**Authors:** Estefanía Sucre-Rosales, Nidal Saleh, Jerôme Lacour, Eric Vauthey

**Affiliations:** a Department of Physical Chemistry, University of Geneva Quai Ernest Ansermet 30 1211 Geneva 4 Switzerland eric.vauthey@unige.ch; b Department of Organic Chemistry, University of Geneva Quai Ernest Ansermet 30 1211 Geneva 4 Switzerland jerome.lacour@unige.ch

## Abstract

Boramidine is a small water-soluble organic fluorophore that was recently introduced as a versatile building block of fluorescent probes. Herein, we show that boramidine is protonated in highly protic solvents. This behaviour explains the surprisingly large difference in the absorption spectrum reported previously when going from an organic to an aqueous environment. Transient absorption measurements reveal that the invariance of the fluorescence spectrum to the environment arises from an excited-state proton transfer to the solvent occurring a few ps after photoexcitation of the protonated boramidine. This photoacidity of boramidine is a further add-on to the polyvalence of this fluorophore.

## Abstract 2

Boramidine, a small organic fluorophore, is protonated in water and highly protic media and behaves as a photoacid upon optical excitation.

## Graphical abstract

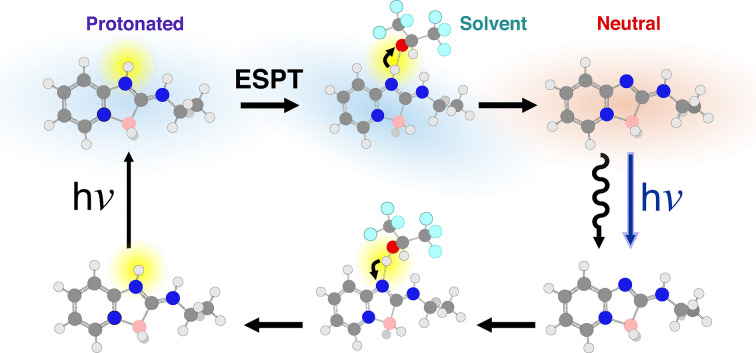

Boramidine (BA, Fig. 1) is a newly introduced boron-based and water soluble chromophore, which was shown to be a potentially powerful building block for the design of fluorescent photoactive compounds.^1^ For example, boramidine-based chiral fluorophores exhibiting significant circularly polarised emission were recently synthesised upon introduction of adjacent chirality axes,^2,3^ or upon addition of a binol tether to induce chiral perturbation.^4^ Interestingly, the electron-rich N atom opposite to the boron atom of BA seems to be a potential site for hydrogen-bond interactions and possibly protonation. If this were the case, BA could also be a powerful building block for the synthesis of excited-state proton-transfer (ESPT) fluorescent probes for various sensing applications.^5–9^ Here, we explore this aspect and investigate the photophysics of BA in protic organic solvents and in aqueous solutions using stationary and time-resolved spectroscopies combined with quantum-chemical calculations. We found that, whereas H-bond interactions are operative in moderately protic solvents, BA is protonated in a highly protic solvent like hexafluoro-2-propanol (HFIP) and in aqueous solutions at neutral and low pH. This explains the surprisingly large blue shift of the main absorption band of BA reported by Lebedev *et al.* when going from organic solvents to water.^1^ Our study also reveals that the protonated BA undergoes ESPT and, thus, acts as a photoacid. These findings open new perspectives for the application of this chromophore in the design of new fluorescent probes.

**Fig. 1:**
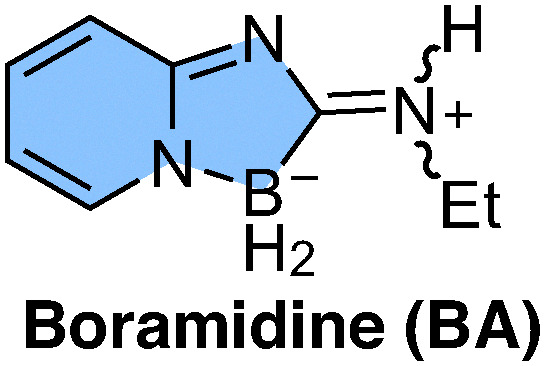
The boramidine chromophore. The *E* and *Z* geometrical isomers are present in a *E*/*Z* ratio of 0.6.

As illustrated in Fig. 2a, the lowest-energy absorption band of BA, assigned to the S_1_ ← S_0_ transition, exhibits a marked solvatochromism in alcohols, shifting continuously to shorter wavelengths upon decreasing the alkyl chain length. This shift correlates with the *α* Kamlet–Taft parameter, which quantifies the H-bond donating ability of the solvent.^10^ The blue shift implies that H-bond interactions with the chromophore are stronger in the ground than in the excited state.^11^ The emission maximum also varies somewhat with the environment, but no clear trend with a solvent property can be detected, in agreement with weaker H-bond interactions in the S_1_ state. In HFIP (*α* = 1.96),^12^ the absorption band peaks below 300 nm, *i.e.*, shifts by more than 5000 cm^−1^ relative to acetonitrile (ACN) (Fig. 2b). This band resembles that measured in water, as also reported by Lebedev *et al.*^1^ Apart from a small red shift of the main band compared to HFIP, the spectrum in water also comprises a weak band around 330 nm, which coincides with the S_1_ ← S_0_ band measured in the other solvents. In sharp contrast with the absorption, the fluorescence spectra in HFIP and water are close to those measured in the other solvents, pointing to the same emissive state. The broadening on the high-energy side of this band in HFIP most probably arises from to the presence of H-bonded excited species in this highly protic solvent. The intense 300 nm absorption band in water was assigned to the S_3_ ← S_0_ transition,^1^ but no explanation for its large intensity compared to that measured in organic solvents was proposed. This interpretation does not really account for the absence of the low-energy S_1_ ← S_0_ band in HFIP. Given the H-bond interactions observed in alcohols, we suspected that the strongly blue-shifted absorption band in HFIP and water could be due to a protonated form of BA.

**Fig. 2:**
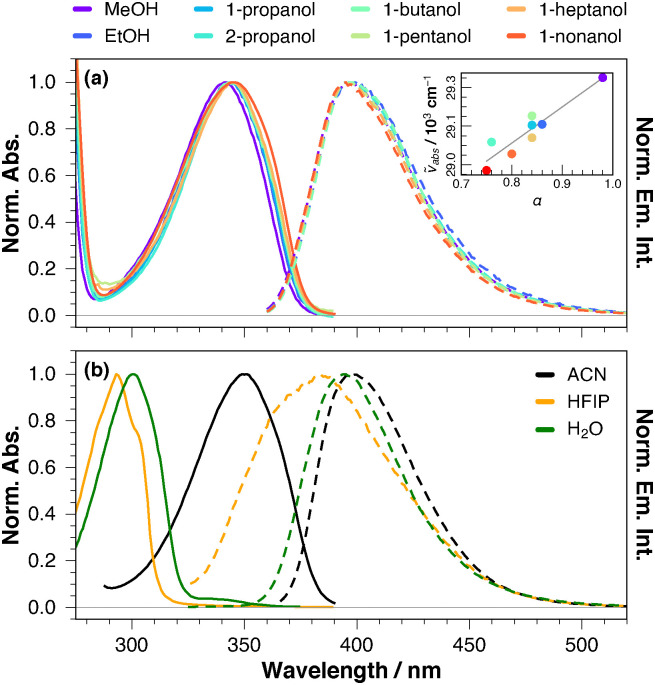
(a) Normalised electronic absorption and emission spectra of BA in alcohols. Inset: Correlation between the absorption maxima and the Kamlet–Taft *α* parameter. (b) Normalised electronic absorption and emission spectra of BA in acetonitrile (ACN), hexafluoroisopropanol (HFIP), and water.

To explore this hypothesis, the absorption and emission spectra of BA were measured in an aqueous 0.1 M Britton–Robinson buffer (BR),^13^ with a pH varying from 5 to 10. As illustrated in Fig. 3, the absorption spectrum at the lowest pH exhibits the 300 nm band and two intense bands at 236 and 217 nm, as measured in neutral aqueous solution. At pH > 7, these three bands decrease, while two new bands around 340 and 260 nm rise concurrently. At pH > 10, the 300 and 236 nm bands are no longer visible. The two isosbestic points around 315 and 245 nm are indicative of the presence of an acid–base equilibrium in the ground state. These results demonstrate unambiguously that the 300 nm band in water is due to the S_1_ ← S_0_ transition of a protonated form of the boramidine, BAH^+^. The same behaviour is observed in an aqueous 0.1 M HEPES (4-(2-hydroxyethyl)-1-piperazineethanesulfonic acid) buffer (Fig. S1a, ESI†). Contrary to absorption, the emission spectrum remains unchanged independently of the pH of the solution (Fig. 3 and Fig. S1a, ESI†). Moreover, the fluorescence excitation spectrum matches the absorption spectrum over the whole pH range investigated, and comprises the bands of both BA and BAH^+^ between pH 8 and 9 (Fig. S1b, ESI†). These observations imply that the fluorescence originates from the non-protonated form of BA. Given the significant oscillator strength of the S_1_ ← S_0_ transition reflected by the large relative intensity of the 300 nm absorption band, BAH^+^ should in principle be as fluorescent as BA. Therefore, the absence of an additional band due to BAH^+^ in the emission spectra recorded at low pH suggests that ultrafast ESPT is taking place.^14–17^

**Fig. 3:**
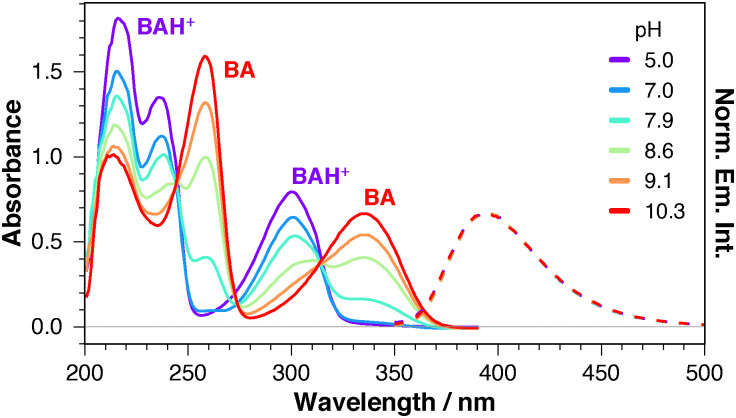
Electronic absorption spectra of BA in Britton–Robinson buffer (0.1 M) at increasing pH values. Normalised fluorescence of BA (300 nm excitation) at pH = 5.0 and pH = 10.3.

Occurrence of ultrafast ESPT upon photoexcitation of BAH^+^ was investigated using transient electronic absorption (TA) spectroscopy in aqueous HEPES buffer at pH 7.5, and in HFIP (p*K*_a_ =9.3 in water)^18^ (Fig. 4 and Fig. S2, S3, ESI†). In both media, the early TA spectra exhibit a broad positive band covering the whole visible region as well as a negative band below 370 nm, which extends beyond the spectral window of the experiment in water. This negative feature transforms in a few ps into another negative band centred around 400 nm in water and 380 nm in HFIP. Meanwhile, the broad positive band narrows and shifts to shorter wavelengths. Afterwards, the TA spectrum remains unchanged and its amplitude decays on the nanosecond timescale, beyond the time window of the experiment. This late spectrum resembles that reported for BA in ACN with the positive band assigned to an S_*n*>1_ ← S_1_ excited state absorption (ESA) and the negative band attributed to the S_1_ → S_0_ stimulated emission (SE) of BA.^2^ In ACN, the S_1_ state of BA was found to decay on the 10 ns timescale mainly *via* fluorescence and inter-system crossing. Perdeuteration of HFIP did not result in significant differences (Fig. S3, ESI†). The negative band below 370 nm is not related to a ground-state bleach feature, because BAH^+^ absorbs only below 320 nm. It can thus be attributed to the S_1_ → S_0_ stimulated emission of BAH^+^*. Based on this, the broad positive band measured at early times is most probably due to an ESA of BAH^+^* as well. Consequently, the early spectral dynamics measured in these two solvents reflect the BAH^+^* → BA* ESPT.

**Fig. 4:**
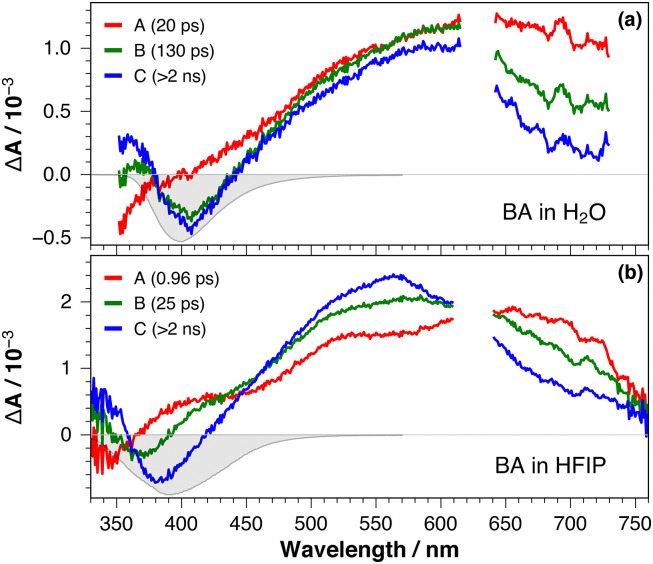
Evolution-associated difference absorption spectra and time constants obtained from a global analysis of the data recorded upon 310 nm excitation of BA in (a) aqueous HEPES buffer and (b) HFIP, assuming a series of three successive exponential steps (A → B → C →), together with the negative stationary stimulated emission spectrum (gray).

To determine the timescale of this process, the TA data were analysed globally assuming a succession of single exponential steps.^19^ A series of three steps was sufficient to obtain good fits, with the evolution-associated difference absorption spectra (EADS) and time constants depicted in Fig. 4a and b. The A → B step in HEPES buffer includes the red shift of the SE band and can, thus, be attributed to ESPT with a 20 ps time constant. The second step is slower and does not lead to significant changes in the SE band. This step is possibly related to some relaxation process occurring after ESPT.

In HFIP, the shift of the SE band takes place during both A → B and B → C steps associated with 1 and 25 ps time constants, respectively (Fig. 4b). Given that the amplitude of the 1 ps band shift is markedly larger than the other, namely 2100 *vs.* 780 cm^−1^, the A → B step is attributed to the ESPT itself, whereas the second one is interpreted as a follow-up relaxation process (*vide infra*). In HFIP-*d*_2_, the dynamics are essentially the same as in HFIP (Fig. S3c, ESI†). This absence of significant deuterium effect points to a low proton-transfer barrier,^20–22^ and a process occurring in the adiabatic regime.^23^ This is consistent with the very short ESPT time constant measured in this solvent. The decay of the ESPT product, BA*, is too slow to be observed here, but should occur similarly to that measured in ACN and lead to the repopulation the ground state.^2^ Contrary to ACN, however, ground-state recovery in aqueous solution and HFIP should be followed by the protonation of BA to BAH^+^, as summarised in Fig. 5.

**Fig. 5:**
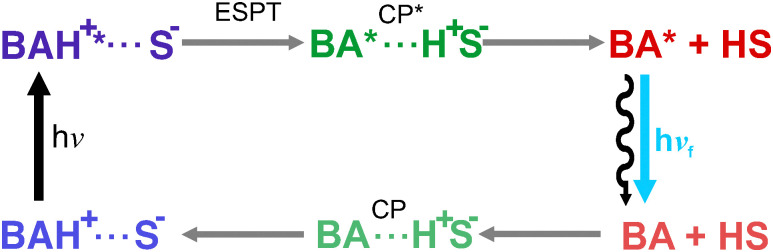
Eigen–Weller model of the excited-state proton transfer to the solvent. For simplity, all equilibria are omitted.

ESPT to the solvent is usually discussed in terms of the Eigen–Weller model that includes an intermediate, a contact pair (CP*), between the excited reactant and product states.^15,17,24–26^ In the present case, CP* should correspond to BA*⋯H^+^, where the leaving proton is only weakly interacting with BA*.^27^ A quantitative analysis of the experimental data using this model would require additional measurements to obtain reliable kinetic parameters and is beyond the scope of this investigation. However, the EADS B obtained from the analysis in HFIP, with the SE band between that of BAH^+^* and BA* could possibly arise from such a contact pair, CP*. This is consistent with the early TA spectra measured in HFIP upon photoexcitation at 320 nm at the red edge of the S_1_ ← S_0_ absorption band of BAH^+^, which show a broad SE band covering the same wavelength range as the SE from both BAH^+^* and BA* (Fig. S4b, ESI†). This suggests that the low-energy side of this intense band could be due to the absorption of loosely protonated or strongly H-bonded molecules, whose photoexcitation leads to the direct population of the CP* state. Such a SE from an intermediate state is not visible in aqueous HEPES buffer. This could be due to the fact that, in this medium, the dissociation of CP* is faster than the ESPT step itself, making the instantaneous population of this intermediate below the detection limit. This is consistent with the slower ESPT in aqueous buffer compared to HFIP, 20 *vs.* 1 ps, and the lower viscosity of water, 1 *vs.* 1.65 cP.

The TA results in HFIP and aqueous buffer reveal the very fast deprotonation of BAH^+^ upon photoexcitation, making this species an efficient photoacid. The p*K*_a_ values of BAH^+^ in both its ground and excited states were determined from stationary electronic absorption and emission measurements as a function of pH in both BR and HEPES buffers (ESI,† Section S2).^28,29^ A p*K*_a_ value of 8.9 ± 0.1 was determined for BAH^+^ in the ground state. For the excited state, a 
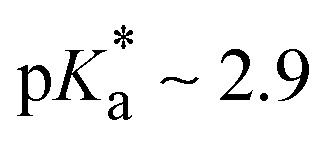
 was estimated using the Förster cycle.^30^ This value is significantly lower than that of the ground state, as expected for a photoacid and is similar to that reported for 2-naphthol which is considered a weak photoacid.^31^

Finally, to better understand the origin of the photoacidity of boramidines, we performed quantum-chemical calculations of BA and BAH^+^ at the density functional theory (DFT) and time-dependent (TD) DFT levels (B3LYP/6-311g++(d,p)). The probable protonation site was determined from the electrostatic potential map of BA (Fig. 6 bottom), which indicates that the highest electronic density can be found at the negatively-charged BH_2_ fragment and at the nitrogen opposite to the boron atom, making this N atom the most likely site for protonation. This is supported by geometry-optimisation calculations, which confirm that this hypothetical structure corresponds to a distinct energy minimum. This protonation site is also consistent with the ^1^H-NMR spectra of BA in DCM-*d*_2_ before and after addition of HFIP to 1% (v/v) (Fig. S6, ESI†).

**Fig. 6:**
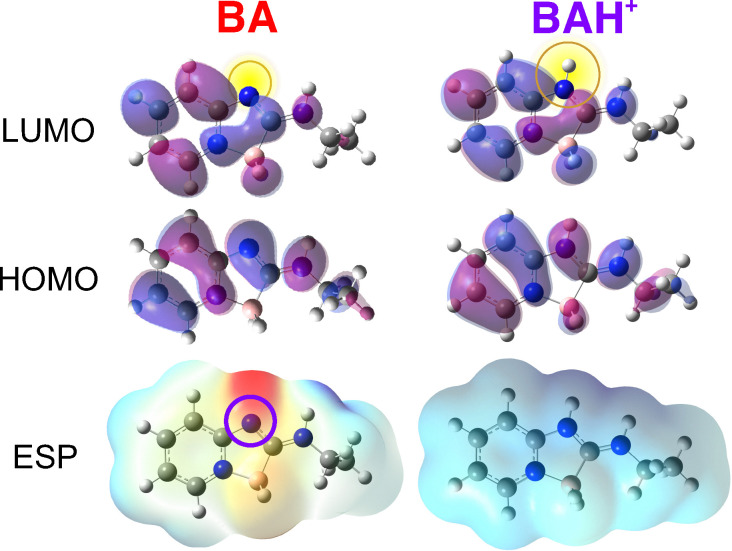
Top: Frontier molecular orbitals of BA and BAH^+^, with the protonation site highlighted in yellow. Bottom: Electrostatic potential (ESP) maps of BA and BAH^+^. The zones with more negative (positive) electrostatic potential are in red (blue).

Although it was not possible to identify the signal corresponding to the labile acidic proton, indirect evidence of protonation can be found in the variation of the *E*/*Z* ratio upon addition of HFIP. In fact, it has been previously shown that the *E*/*Z* ratio of boramidines remains essentially constant under neutral conditions.^2,4^ However, in the present case with ∼10 molar equiv. of HFIP, the *E*/*Z* ratio increases from 0.6 to 4.1, which is consistent with the observation of the *E* isomer only upon electrophilic substitution of the nitrogen at the C=N amidine bond (Fig. S7, ESI†).^1^

Furthermore, the higher energy of the S_1_ ← S_0_ transition of BAH^+^ relatively to BA is qualitatively well reproduced by TD-DFT calculations (Fig. S5, ESI†). For both species, this excitation is dominated by a one-electron HOMO to LUMO transition. As illustrated in Fig. 6 top, this transition results in a strong decrease of the electronic density on the N atom where protonation takes place. The resulting weakening of the N–H^+^ bond is at the origin of the photoacidity of BAH^+^. The high electronic density on the N atom is restored after the decay of BA* to the ground state, and protonation occurs again.

This investigation reveals that boramidine, a compact water-soluble fluorophore, exists in a protonated form in neutral aqueous solution. This explains the large differences in the absorption spectrum reported earlier when going from an organic solvent to water.^1^ On the other hand, the emission spectrum is essentially independent of the solvent and pH in aqueous media. This is due to the photoacidity of the protonated boramidine, whose photoexcitation is followed by an excited-state proton transfer to the solvent occurring on the ps timescale. Consequently, the fluorescence band is shifted by more than 7000 cm^−1^ relative to the absorption. This photoacidity is an additional feature of boramidines that further increases its polyvalence as a platform for the design of fluorescent probes.

## Conflicts of interest

There are no conflicts to declare.

## Supplementary Material

CC-061-D5CC02043C-s001

## Data Availability

All data can be downloaded from https://doi.org/10.26037/yareta:r7o5q4mlo5eshmlmjuvjklfa7u.
