# Supplementary material for: Boramidine: a boron-based photoacidic fluorophore
Source: Chem Commun (Camb). 2025 May 15;61(49):8863–6. doi: 10.1039/d5cc02043c (PMC12093955; doi:10.1039/d5cc02043c)
Supplement: CC-061-D5CC02043C-s001 [file CC-061-D5CC02043C-s001.pdf]

Supplementary Information (SI) for ChemComm.
This journal is © The Royal Society of Chemistry 2025

Boramidine: A boron-based photoacidic fluorophore

Estefanía Sucre-Rosales,[a] Nidal Saleh,[b] Jérôme Lacour,*[b] and Eric Vauthey*[a]

[a]*Department of Physical Chemistry, University of Geneva, 30 Quai Ernest-Ansermet, CH-1211 Geneva 4, Switzerland. E-mail: eric.vauthey@unige.ch*

[b] *Department of Organic Chemistry, University of Geneva, 30 Quai Ernest-Ansermet, CH-1211 Geneva 4, Switzerland. E-mail: jerome.lacour@unige.ch*

Contents

S1 Experimental Details **3**
S1.1 Samples . . . 3
S1.2 Stationary spectroscopy . . . 3
S1.3 Transient absorption spectroscopy . . . 3
S1.4 Quantum chemical calculations . . . 3

S2 Calculation of the pK_a of BAH^+ in the ground and excited states **4**

S3 Additional results **5**
S3.1 Stationary electronic spectra . . . 5
S3.2 Transient electronic absorption spectra . . . 6
S3.3 Quantum-chemical calculations . . . 9
S3.4 ^{1}H-NMR measurements . . . 10

List of Figures

S1 **BA** in 0.1 M HEPES buffer at selected pH values: a) Absorption (continuous lines) and fluorescence spectra (λ_{exc}= 355 nm, dashed lines. The different fluorescence intensities reflect the different absorbance at the excitation wavelength). b) Absorption (continuous lines) and excitation (dashed lines) spectra measured at 400 nm. The differences between the absorption and excitation spectra are attributed to the absence of a reliable correction curve in the UV region. . . 5

S2 a) Transient absorption spectra recorded at various time delays after 310 nm excitation of **BA** in aqueous HEPES buffer at pH=7.5 and b) evolution-associated difference spectra and time constants obtained from a global analysis assuming a series of three successive exponential steps (A→B→C→). 6

S3 Transient absorption spectra recorded at various time delays after 310 nm excitation of **BA** in (a) HFIP and (b) HFIP-d_2. (c) Time dependenc e of the transient absorption at the maximum of the stimulated emission band (390 nm) measured with **BA** in HFIP and in HFIP-d_2. 7

S4 Transient absorption spectra recorded at various time delays after excitation at (a) 310 nm and (b) 320 nm (Red Edge Excitation) of **BA** in HFIP and (c,d) evolution-associated difference spectra and time constants obtained from a global analysis assuming a series of three successive exponential steps (A→B→C→). Given the similarity of EADS B and C in panel d, the error on the B→C time constant is very large. 8

S5 Comparison of the calculated (B3LYP/6-311G++(d,p) in vacuum, broadened using Gaussian functions with a half-width at half-maximum of 0.33 eV) and experimental (0.1 M Britton–Robinson buffer) stationary electronic absorption spectra of **BA** and **BAH**$^+$ 9

S6 ^{1}H-NMR spectra of non-protonated (upper, red) and protonated (bottom, blue) boramidine in DCM and DCM$-d_2$ + 50 μL of HFIP. The acid base equilibria is shown on the right. The signals corresponding to DCM, HFIP and water are inside green, gray and blue squares, respectively. . . . 10

S7 Structures of the N-electrophile-substituted derivatives of boramidines **[2b-Et]BF$_4$**, **2b-Et** and **2b-BF$_3$**, which are only present as *E* isomers (the numbering is the same as that in ref. 1). 11

S1 Experimental Details

S1.1 Samples

All solvents were of HPLC and/or spectroscopic grade, and were used as received. Nanopure (mili-Q) water was also used. BA was synthesized and purified as follows.

In a flame-dried Schlenk tube and under nitrogen atmosphere, freshly prepared $[Me_3NBH_2CNEt]BF_4$ (4 mmol, 1.5 equiv) in 5 mL of dry CH_2Cl_2 (DCM) was added to aminopyridine (188 mg, 2 mmol) dissolved in 10 mL of dry DCM. The solution was stirred at room temperature and the reaction was monitored by ^{1}H-NMR and ESI-MS. After 24 h the solution was diluted with DCM (10 mL) and washed with brine (2 x 10 mL), then extracted with saturated aq. Na_2CO_3. The organic phase was dried over anhydrous Na_2SO_4 and the solvent was removed under reduced pressure. The crude product was purified by silica gel flash chromatography (DCM:MeOH 97:3) to give the 230 mg (71%, white precipitate) of the desired product as a mixture of E/Z isomers (2:1 ratio). The NMR data were similar to those reported in the literature[1].

^{1}H-NMR (400 MHz, DCM$-d_6$) δ 8.00 (d, J = 6.7 Hz, 0.67H, major), 7.94 (d, J = 6.7 Hz, 0.33H, minor) 7.68 – 7.59 (m, 1H), 7.09 (d, J = 8.6 Hz, 0.33H, minor), 7.04 (d, J = 8.6 Hz, 0.67H, major), 6.76 (ddd, J = 7.1, 5.9, 1.2 Hz, 0.67H, major), 6.72 (ddd, J = 7.1, 5.8, 1.2 Hz, 0.33H, minor), 3.55 – 3.47 (m, 0.66H, minor), 3.32 (q, J = 7.2 Hz, 1.34H, major), 3.24 – 2.29 (m, 2H), 1.23 (m, 3H, major + minor).

The Britton–Robinson Buffer was prepared following the protocol reported in ref. 2.

S1.2 Stationary spectroscopy

All measurements were performed in 1 cm cuvettes. The pH was determined with a Mettler-Toledo® pH meter. The stationary electronic absorption spectra were measured using a Cary 50 spectrometer, while the stationary emission spectra were recorded on a Horiba FluoroMax-4 spectrofluorometer. Emission spectra were corrected to account for the detector and grating responses. Excitation spectra were corrected according to the lamp profile. However, the observed difference between excitation and absorption spectra were attributed to an unreliable correction curve in the UV region. The stimulated emission spectra were calculated by multiplying the spontaneous fluorescence intensity by λ^4, where λ is the wavelength.[3]

S1.3 Transient absorption spectroscopy

Transient absorption measurements were performed using a setup described in detail in ref. 4. Excitation was performed at 310 and 320 nm, by doubling the output of a TOPAS-Prime with a NirUVis frequency mixer (both from Light Conversion), itself seeded by the output of a 5 kHz Ti:Sapphire amplified system (Spectra Physics, Solstice Ace). Linearity of the transient absorption signal with the pump intensity was checked prior to the measurement. The polarization of the pump pulses was set to magic angle relative to the white-light pulses. All samples were kept under nitrogen flow during the entire duration of the measurement (around 1.5-2 h). Global analysis was performed using a homemade Matlab® script.[5]

S1.4 Quantum chemical calculations

Geometry optimizations were carried out in vacuum using the Gaussian 16, rev. A.03 software,[6] with the hybrid B3LYP functional and the 6-311G++(d,p) basis set. Time-dependent density functional theory (TD-DFT) calculations were performed at the same level of theory. The electronic absorption spectra were calculated using a Gaussian line broadening function with a half width at half maximum of 0.333 eV.

S2 Calculation of the pK_a of BAH^+ in the ground and excited states

We used the pH-dependent absorbance in both Britton-Robinson and HEPES buffers, and the extinction coefficients of **BAH**$^+$ and **BA**, according to equation S1[7]:

$$\begin{cases} A_{BAH^+} = \varepsilon_{BAH^+,\lambda_{BAH^+}}[BAH^+] + \varepsilon_{BAH^+,\lambda_{BA}}[BA] \\ A_{BA} = \varepsilon_{BA,\lambda_{BAH^+}}[BAH^+] + \varepsilon_{BA,\lambda_{BA}}[BA] \end{cases} \tag{S1}$$

where $\varepsilon_{BAH^+,\lambda_{BAH^+}}$ and $\varepsilon_{BAH^+,\lambda_{BA}}$ are the extinction coefficients of **BAH**$^+$ at the maximum absorption wavelength of the spectrum of the protonated and non-protonated species, respectively; $\varepsilon_{BA,\lambda_{BAH^+}}$ and $\varepsilon_{BA,\lambda_{BA}}$ are the extinction coefficients of **BA** at the maximum absorption wavelength of the protonated and deprotonated species, respectively; and finally, $[BAH^+]$ and $[BA]$ are the concentrations of **BAH**$^+$ and **BA**. Then, once the pH-dependent magnitudes of **BAH**$^+$ and **BA** are calculated, the pK_a of the compound can be found using the Henderson–Hasselbalch equation (S2) and taking its average:

$$pK_a = pH - \log \frac{[BA]}{[BAH^+]} \tag{S2}$$

This method yielded a pK_a of **BA** in the ground state of 8.9 ± 0.1.

For the excited state, the pK_a^* was estimated using the Förster cycle:[8]

$$pK_a^* \approx 2.07 \times 10^{-3}(\Delta\tilde{\nu}_{BAH^+} - \Delta\tilde{\nu}_{BA}), \tag{S3}$$

with the S_1-S_0 energy gaps ($\Delta\tilde{\nu}$ in cm^{-1}) of the protonated and non-protonated species obtained from the average absorption and fluorescence energy gaps. Since the ultrafast proton transfer to the solvent prevents detection of stationary emission from **BAH**$^{+*}$, the fluorescence maximum of this species was estimated from the SE band observed in the early TA spectra. A p$K_a^* \sim 2.9$ was obtained in the Britton–Robinson buffer, significantly lower than the ground state, as expected for a photoacid. The same value was obtained when using the higher energy absorption bands of the protonated and non-protonated forms, peaking at 236 and 259 nm respectively, as well as when working in HEPES buffer.

S3 Additional results

S3.1 Stationary electronic spectra

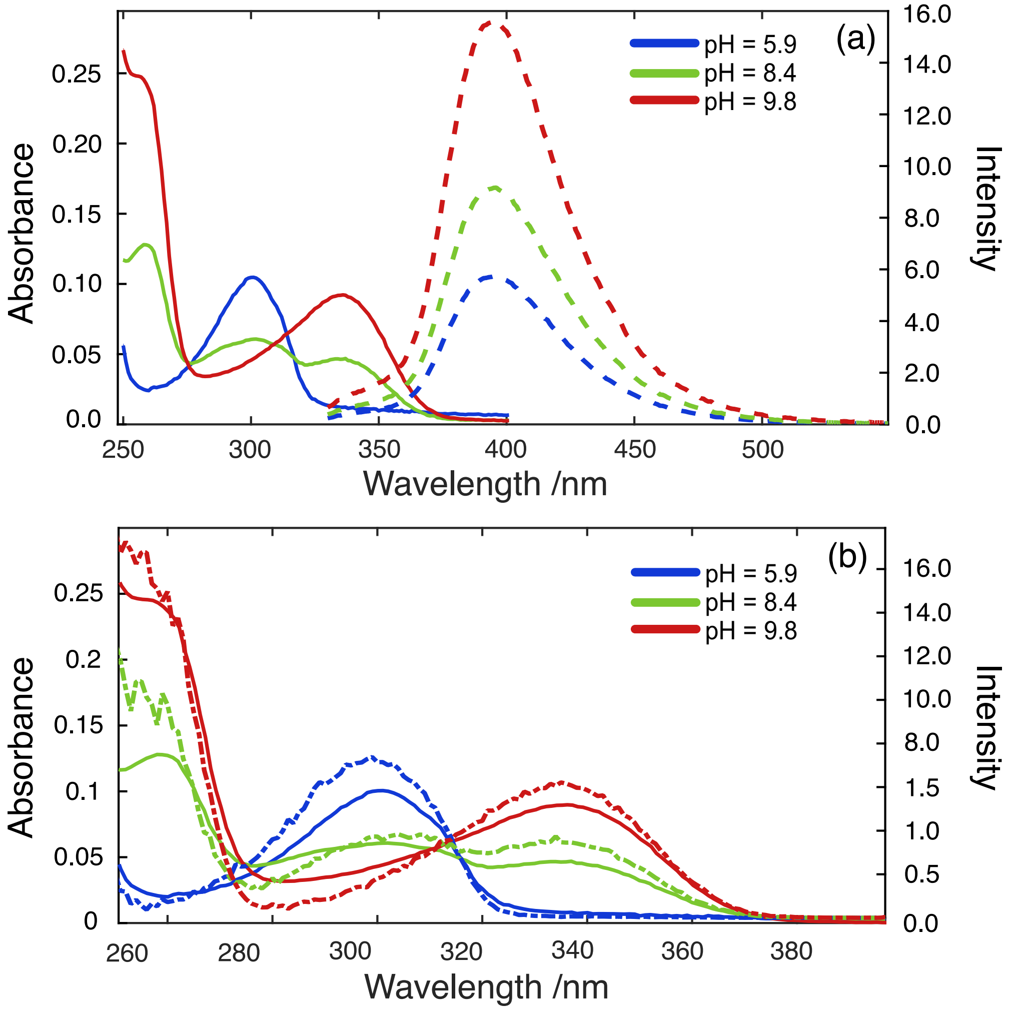

Figure S1 BA in 0.1 M HEPES buffer at selected pH values: a) Absorption (continuous lines) and fluorescence spectra (λ_{exc}= 355 nm, dashed lines. The different fluorescence intensities reflect the different absorbance at the excitation wavelength). b) Absorption (continuous lines) and excitation (dashed lines) spectra measured at 400 nm. The differences between the absorption and excitation spectra are attributed to the absence of a reliable correction curve in the UV region.

S3.2 Transient electronic absorption spectra

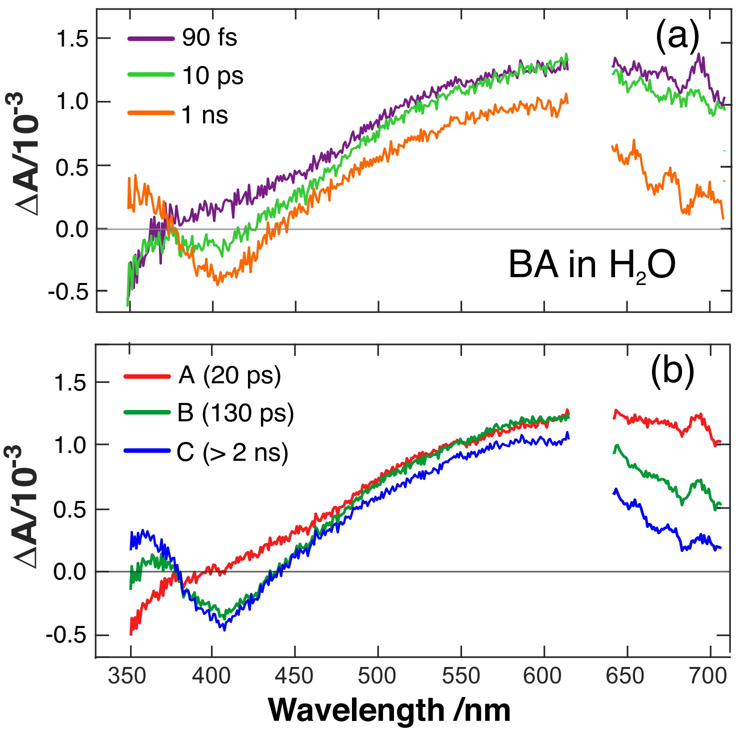

Figure S2 a) Transient absorption spectra recorded at various time delays after 310 nm excitation of **BA** in aqueous HEPES buffer at pH=7.5 and b) evolution-associated difference spectra and time constants obtained from a global analysis assuming a series of three successive exponential steps (A→B→C→).

.

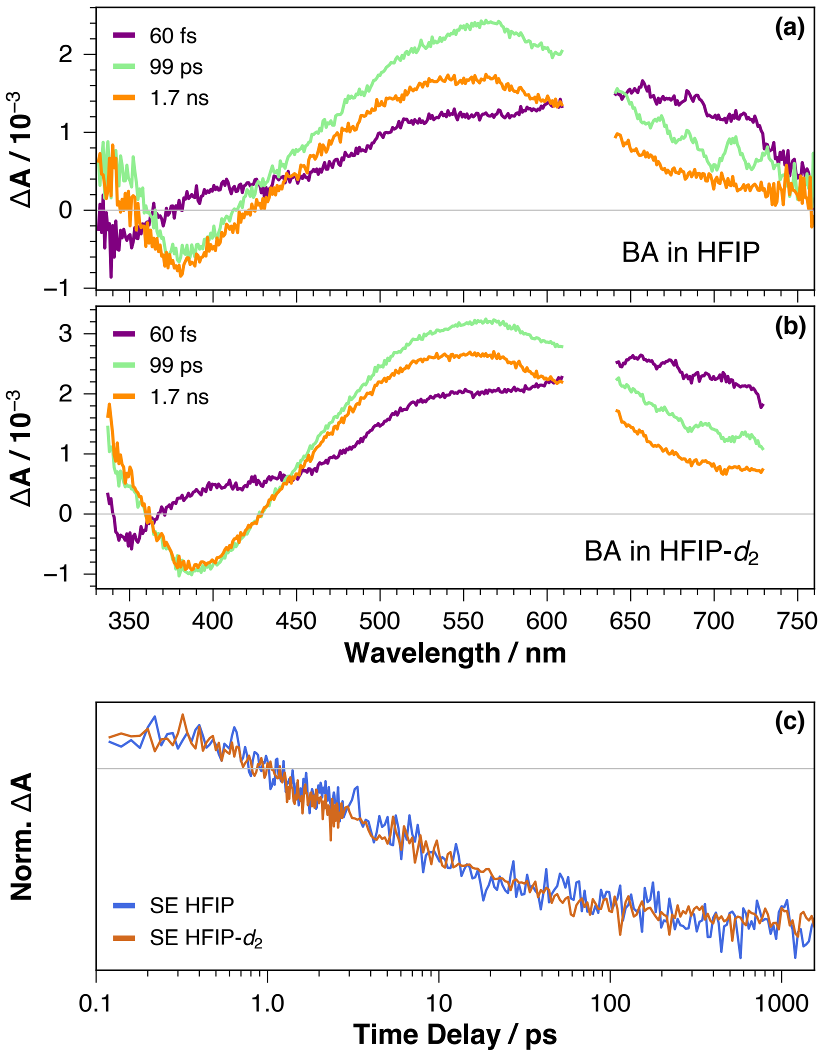

Figure S3 Transient absorption spectra recorded at various time delays after 310 nm excitation of **BA** in (a) HFIP and (b) HFIP-d_2. (c) Time dependenc e of the transient absorption at the maximum of the stimulated emission band (390 nm) measured with **BA** in HFIP and in HFIP-d_2.

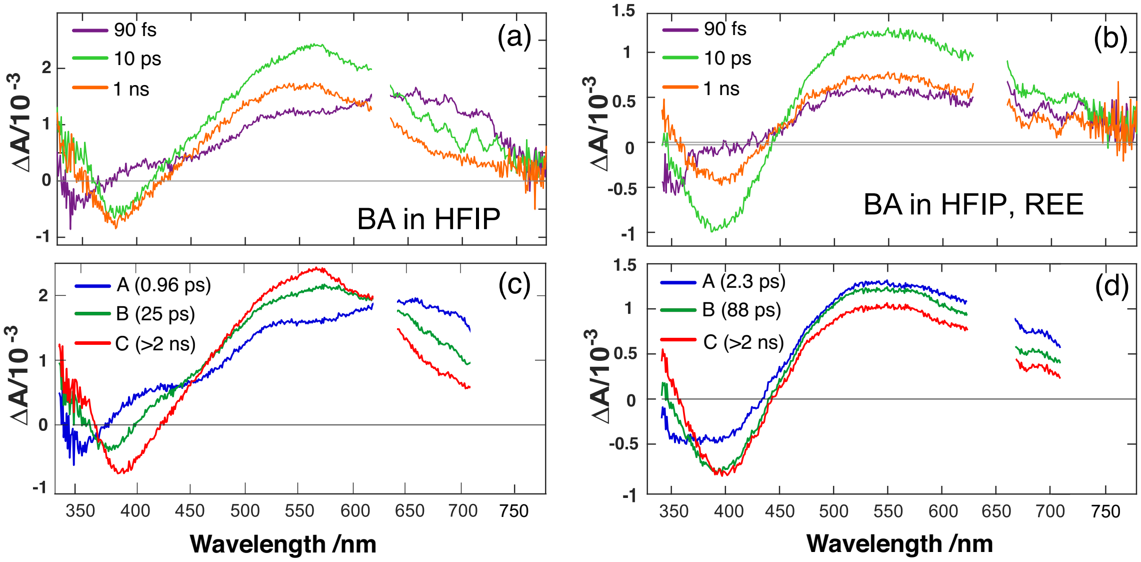

Figure S4 Transient absorption spectra recorded at various time delays after excitation at (a) 310 nm and (b) 320 nm (Red Edge Excitation) of **BA** in HFIP and (c,d) evolution-associated difference spectra and time constants obtained from a global analysis assuming a series of three successive exponential steps (A→B→C→). Given the similarity of EADS B and C in panel d, the error on the B→C time constant is very large.

.

S3.3 Quantum-chemical calculations

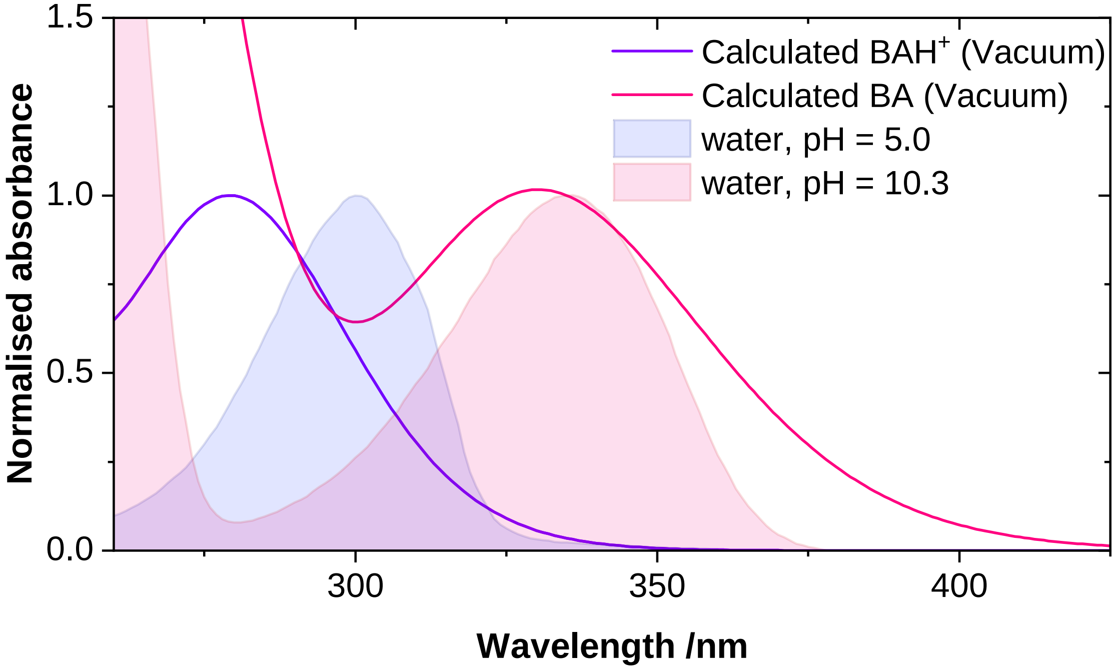

Figure S5 Comparison of the calculated (B3LYP/6-311G++(d,p) in vacuum, broadened using Gaussian functions with a half-width at half-maximum of 0.33 eV) and experimental (0.1 M Britton–Robinson buffer) stationary electronic absorption spectra of **BA** and **BAH**$^{+}$

.

S3.4 ^{1}H-NMR measurements

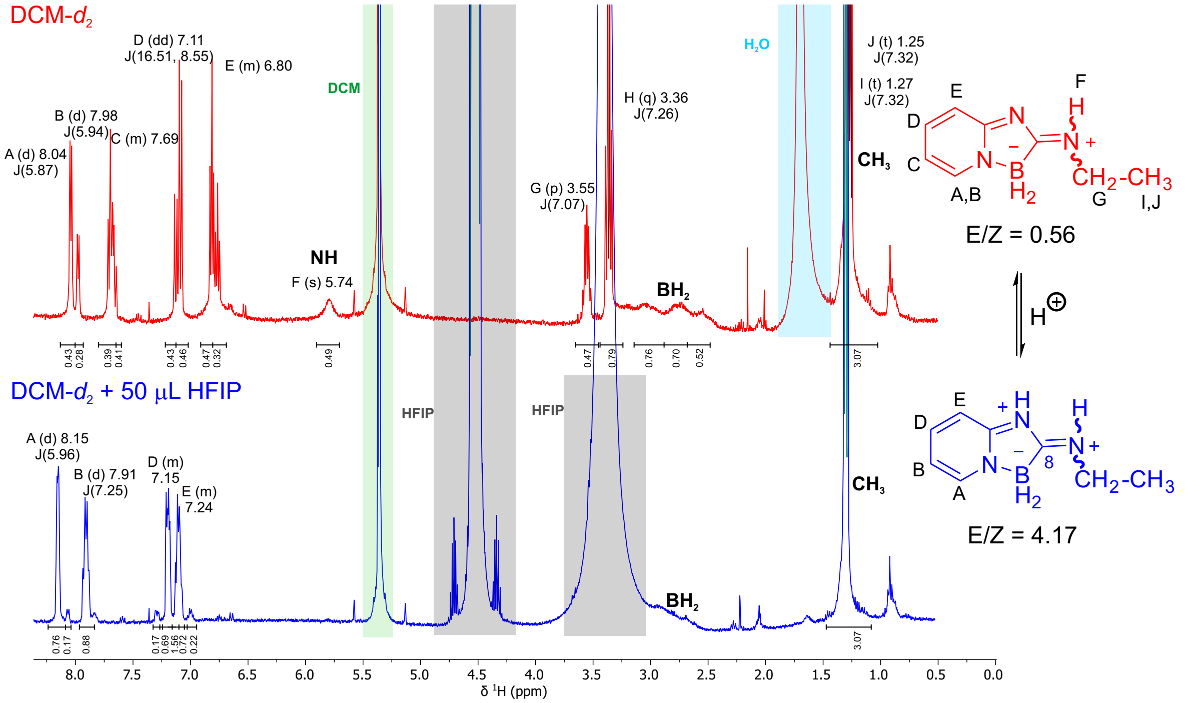

Figure S6 ^{1}H-NMR spectra of non-protonated (upper, red) and protonated (bottom, blue) boramidine in DCM and DCM$-d_2$ + 50 μL of HFIP. The acid base equilibria is shown on the right. The signals corresponding to DCM, HFIP and water are inside green, gray and blue squares, respectively.

To characterize **BAH**$^+$, its ^{1}H-NMR spectrum in DCM$-d_2$ and HFIP was compared it with that of **BA** in DCM (Figure S6). The ^{1}H-NMR spectrum of **BA** in DCM$-d_2$ is consistent with that previously reported in $CDCl_3$,[1] with only a minor upfield shift due to the higher polarity of DCM. Additionaly, the E/Z ratio of 0.6 coincides with that reported. Upon addition of about 10 molar equiv. of HFIP, this ratio is increased to 4.1 and the aromatic lines are shifted downfield, pointing to the presence of a formal positive charge which deshields the aromatic ring protons. As expected, the alkyl lines (which constitute an isolated spin system) remain unaffected upon protonation. The presence of E as the major isomer is consistent with other N-electrophile-substituted derivatives, namely **[2b-Et]BF$_4$**, **2b-Et** and **2b-BF$_3$** (Figure S7), which were reported to be only present as E isomers.[1] This suggests that protonation occurs at the nitrogen of the C=N amidine bond. Nevertheless, the line corresponding to this proton cannot be observed, since is a labile proton and it can rapidly be exchanged. The same happens with the line corresponding to the N-H proton of the C=NH$^+$−Et (signal F, $\delta = 5.74$), which is clearly visible in the absence of HFIP.

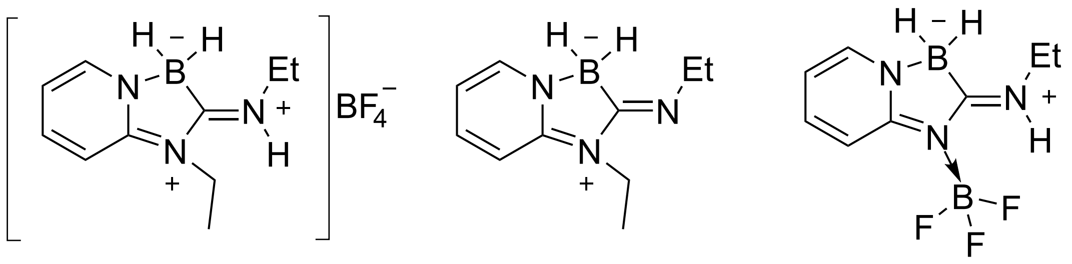

[2b-Et]BF$_4$ only *E* | **2b-Et** only *E* | **2b-BF$_3$** only *E*

Figure S7 Structures of the N-electrophile-substituted derivatives of boramidines **[2b-Et]BF$_4$**, **2b-Et** and **2b-BF$_3$**, which are only present as *E* isomers (the numbering is the same as that in ref. 1).

References

[1] Y. Lebedev, C. Apte, S. Cheng, C. Lavigne, A. Lough, A. Aspuru-Guzik, D. S. Seferos and A. K. Yudin, *J. Am. Chem. Soc.*, 2020, **142**, 13544–13549.

[2] V. Mongay, Carlos, and Cerda, *Ann. Chim.*, 1974, **64**, 409–412.

[3] A. V. Deshpande, A. Beidoun, A. Penzkofer and G. Wagenblast, *Chem. Phys.*, 1990, **142**, 123–131.

[4] J. S. Beckwith, A. Aster and E. Vauthey, *Phys. Chem. Chem. Phys.*, 2022, **24**, 568–577.

[5] R. J. Fernández-Terán, E. Sucre-Rosales, L. Echevarria and F. E. Hernández, *J. Chem. Educ.*, 2022, **99**, 2327–2337.

[6] M. J. Frisch, G. W. Trucks, H. B. Schlegel, G. E. Scuseria, M. A. Robb, J. R. Cheeseman, G. Scalmani, V. Barone, G. A. Petersson, H. Nakatsuji, X. Li, M. Caricato, A. V. Marenich, J. Bloino, B. G. Janesko, R. Gomperts, B. Mennucci, H. P. Hratchian, J. V. Ortiz, A. F. Izmaylov, J. L. Sonnenberg, D. Williams-Young, F. Ding, F. Lipparini, F. Egidi, J. Goings, B. Peng, A. Petrone, T. Henderson, D. Ranasinghe, V. G. Zakrzewski, J. Gao, N. Rega, G. Zheng, W. Liang, M. Hada, M. Ehara, K. Toyota, R. Fukuda, J. Hasegawa, M. Ishida, T. Nakajima, Y. Honda, O. Kitao, H. Nakai, T. Vreven, K. Throssell, J. Montgomery, J. A., J. E. Peralta, F. Ogliaro, M. J. Bearpark, J. J. Heyd, E. N. Brothers, K. N. Kudin, V. N. Staroverov, T. A. Keith, R. Kobayashi, J. Normand, K. Raghavachari, A. P. Rendell, J. C. Burant, S. S. Iyengar, J. Tomasi, M. Cossi, J. M. Millam, M. Klene, C. Adamo, R. Cammi, J. W. Ochterski, R. L. Martin, K. Morokuma, O. Farkas, J. B. Foresman and D. J. Fox, *Gaussian 16, Revision A.03*, 2016, `http://gaussian.com/`.

[7] S. W. Tobey, *J. Chem. Educ.*, 1958, **35**, 514.

[8] Z. R. Grabowski and W. Rubaszewska, *J. Chem. Soc., Faraday Trans. 1*, 1977, **73**, 11–28.
